# It Is Not Just the FEV1 That Matters, but the Personal Goals We Reach Along the Way: Qualitative, Multicenter, Prospective, Observational Study

**DOI:** 10.2196/29218

**Published:** 2021-10-20

**Authors:** Martinus C Oppelaar, Lara S van den Wijngaart, Peter J F M Merkus, Ellen A Croonen, Cindy A C Hugen, Marianne L Brouwer, Annemie L M Boehmer, Jolt Roukema

**Affiliations:** 1 Department of Pediatric Pulmonology Amalia Children's Hospital Radboud University Medical Center Nijmegen Netherlands; 2 Department of Pediatrics Canisius Wilhelmina Hospital Nijmegen Netherlands; 3 Department of Pediatrics Spaarne Gasthuis Haarlem Netherlands

**Keywords:** eHealth, asthma, pediatrics, telemonitoring, lung function tests, lung function, spirometry, home monitoring, mHealth, app, smartphone, asthma control, child, outpatients, remote consultations, quality improvement, patient care management, telemetry, application, FEV1, pulmonary care

## Abstract

**Background:**

The COVID-19 pandemic has boosted the use of forced expiratory volume in 1 second (FEV_1_) telemonitoring in pediatric asthma, but a consensus on its most efficient and effective implementation is still lacking. To find answers, it is important to study how such an intervention is perceived, experienced, and used by both patients and health care professionals (HCPs).

**Objective:**

The aim of this study was to provide perspectives on how FEV_1_ home monitoring should be used in pediatric asthma.

**Methods:**

This is a qualitative, multicenter, prospective, observational study which included patients with asthma aged 6-16 and HCPs. Primary outcomes were results of 2 surveys that were sent to all participants at study start and after 3-4 months. Secondary outcomes consisted of FEV_1_ device usage during 4 months after receiving the FEV_1_ device.

**Results:**

A total of 39 participants (26 patients and 13 HCPs) were included in this study. Survey response rates were 97% (38/39) at the start and 87% (34/39) at the end of the study. Both patients and HCPs were receptive toward online FEV_1_ home monitoring and found it contributive to asthma control, self-management, and disease perception. The main concerns were about reliability of the FEV_1_ device and validity of home-performed lung function maneuvers. FEV_1_ devices were used with a median frequency of 7.5 (IQR 3.3-25.5) during the 4-month study period.

**Conclusions:**

Patients and HCPs are receptive toward online FEV_1_ home monitoring. Frequency of measurements varied largely among individuals, yet perceived benefits remained similar. This emphasizes that online FEV_1_ home monitoring strategies should be used as a means to reach individual goals, rather than being a goal on their own.

## Introduction

The primary aim of asthma care is to reach and maintain asthma control by early recognition and treatment of pulmonary exacerbations (PEx) [1]. In children with asthma, objective measures to aid patients, caregivers, and health care professionals (HCPs) in detecting pulmonary deterioration are crucial [1-3]. Many objective measures have been studied and proposed, but the forced expiratory volume in 1 second (FEV_1_) measured by spirometry remains undefeated as both an objective measure of pulmonary deterioration and a criterion for defining PEx [4-6]. The limitation of FEV_1_ is that it is usually only measured during scheduled outpatient clinic visits and not preceding asthma exacerbations. As a response to the COVID-19 lockdowns, many hospitals have reduced their outpatient clinic capacities which further decreased our ability to timely recognize PEx. Additional unplanned outpatient visits during symptoms were also harder to schedule because of COVID-19 measures in hospitals. It is therefore no surprise that telemonitoring, including FEV_1_ home measurements, has become more popular during the COVID-19 pandemic [7-9].

The value of FEV_1_ telemonitoring has been a subject of debate since portable spirometers became available. Although generally accepted as a feasible intervention in children with limited disease perception, concerns regarding the reliability of the measurements persist, and studies have failed to convincingly show an added value of FEV_1_ home monitoring in general asthma care [1,4,10-15]. Most of these studies used strict monitoring regimes in which patients measure their FEV_1_ daily in order to reduce PEx and health care consumption. As a result, monitoring adherence declined, and HCPs were left with mounts of—mostly irrelevant—FEV_1_ data and eventually the primary objectives were not reached [1,12-15]. This raises the question of what role FEV_1_ telemonitoring should play in pediatric asthma. With the currently rapidly accelerating interests in FEV_1_ telemonitoring, it is even more important to develop perspectives on this topic.

This study aimed to develop new perspectives on how to use FEV_1_ telemonitoring in the future of pediatric asthma care. To achieve this we combined FEV_1_ home monitoring with an online eHealth platform [16-18]. Our main research question was “How do patients, their parents, and HCPs want to make use of FEV_1_ home monitoring?” Patients who already used the eHealth platform in regular pediatric asthma care to monitor asthma control with questionnaires received FEV_1_ monitoring devices which were integrated in the platform. To realistically reflect a regular pediatric care setting, no fixed measurement schedules were used and patients themselves kept responsibility on how often they measured their lung function. Expectations and experiences of both patients and HCP were studied, as well as FEV_1_ device usage.

## Methods

**Figure 1:**
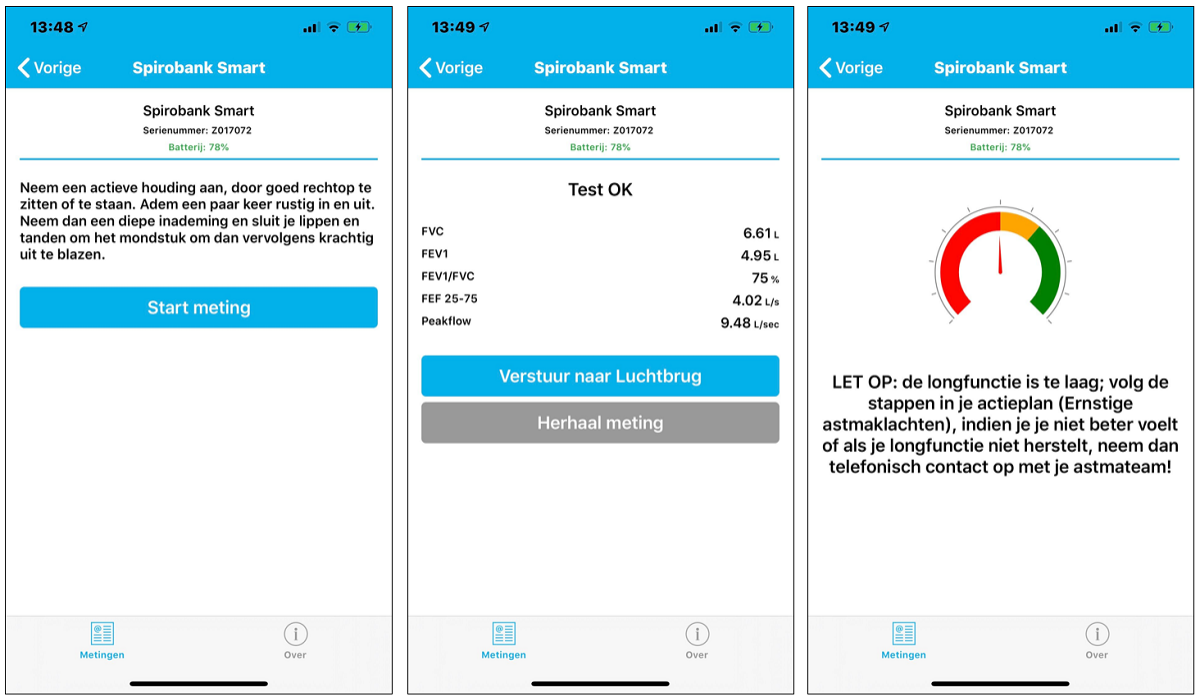
Screenshots of the instruction screen (left panel), measurement screen (middle panel) and feedback screen (right panel) of the smartphone app. The text in the instruction screen provides a short description on how to use the FEV_1_ device appropriately. The measurement screen shows a successfully performed measurement with buttons to upload (upper) or repeat (lower) the measurement. The feedback in the feedback screen is based on individual thresholds. Language is in Dutch.

**Figure 2:**
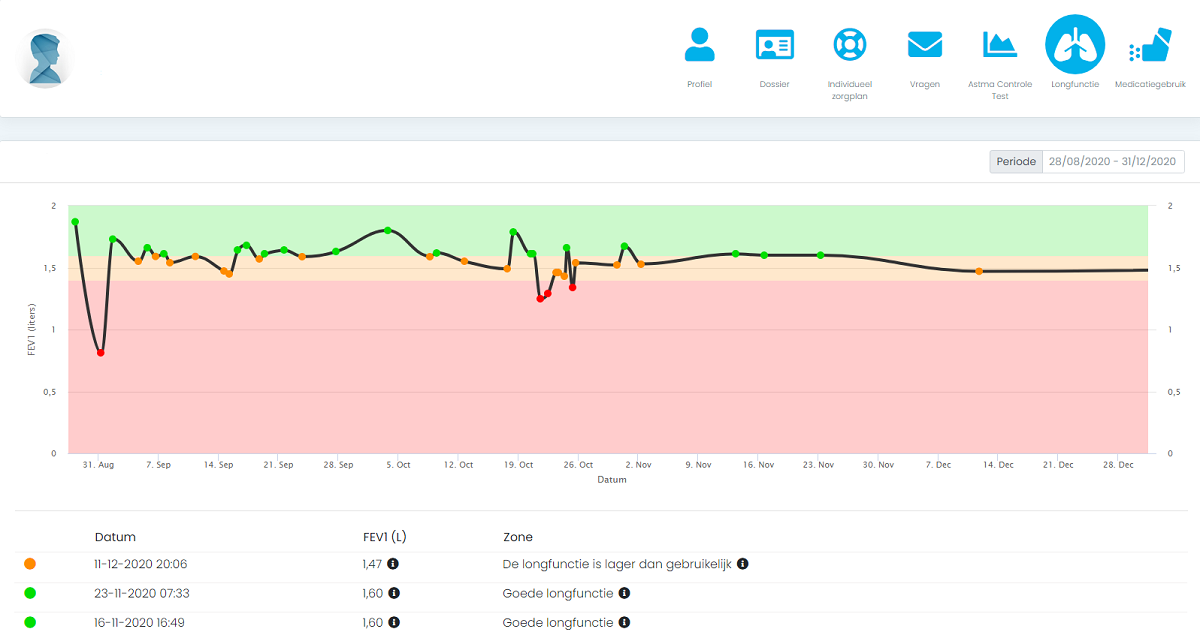
Screenshot of the graphical presentation of FEV_1_ measurements on the online eHealth platform of one of the participants. Language is in Dutch. Colors represent individual color zones (green, orange, red).

This was a qualitative, multicenter, prospective, observational study on FEV_1_ home monitoring combined with an online eHealth platform for 4 months. The eHealth platform is used in regular pediatric asthma care to monitor asthma control using the validated (childhood) Asthma Control Test ([C]-ACT), and to support self-management with personalized online asthma action plans [19,20]. Details of the eHealth platform have been published previously [16-18]. For this study the platform was expanded with a module for FEV_1_ home measurements. FEV_1_ measurements were performed with the Spirobank Smart and automatically uploaded to the online eHealth platform with a smartphone app [21]. The smartphone app was available on both Google Play and the Apple App Store. Participants could log in to the smartphone app with the same credentials as used for the online eHealth platform. After pairing with the FEV_1_ device once, participants could use the app to perform measurements. Measured values were automatically sent to the app via Bluetooth and could be uploaded directly to the online eHealth platform (Figure 1). In addition to FEV_1_, the Spirobank Smart devices measured the forced vital capacity (FVC), FEV_1_-to-FVC ratio (FEV_1_/FVC), peak expiratory flow (PEF), and forced expiratory flow at 25%-75% (FEF25-75). Automated feedback was given for each measurement. A green, orange, or red tag was provided based on individual thresholds depending on the personal best value of FEV_1_ of that patient. A green tag prompted encouraging feedback and required no intervention, while an orange or red tag (ie, usually below 80% of the patient’s best value) prompted an intervention based on the personal online asthma action plan of the patient. A red tag also sent an automatic email notification to the treating HCPs which they could follow-up on if deemed necessary. FEV_1_ values were plotted over time in the online eHealth platform (Figure 2). The FEV_1_ is the main lung function outcome on the eHealth platform, but additional values were visible when selecting specific measurements on the platform. During this study patients kept the responsibility on how often they used their devices, and no instructions were given on how often they needed to measure their FEV_1_.

Participants were recruited during outpatient visits in specialized asthma clinics from a university hospital (Radboudumc, Nijmegen, the Netherlands) and 2 general hospital (Canisius Wilhelmina Hospital, Nijmegen, the Netherlands and Spaarne Gasthuis, Haarlem, the Netherlands). Participants were eligible for inclusion if they had a doctor’s diagnosis of asthma based on the Global Initiative for Asthma (GINA) criteria, were aged 6-18 years, and already used the online eHealth platform for regular pediatric asthma care [1]. All parents and participants aged ≥12 years had documented verbal consent for the anonymized use of their data. The local ethical committee waived approval of the Medical Research Involving Human Subjects Act (WMO) considering the negligible burden of this study and the absence of imposed risks.

The primary outcome included survey results of patients (or their parents in case of young patients) and HCPs before and 3-4 months after the introduction of FEV_1_ devices. We used modified validated research questionnaires originally designed by Grol et al [22] to gather information from 5 perspectives: the receptiveness for using innovations in health care, the perceived contribution of online FEV_1_ home monitoring to asthma care, the perceived contribution of online FEV_1_ home monitoring to patient self-management, the user-friendliness of the service, and the possible undesired effects. The surveys consisted of 21-25 questions of which most were to be answered with a 5-item Likert scale (strongly disagree to strongly agree). The online survey also asked participants to comment on experienced benefits and disadvantages, and to provide suggestions to improve the service. Survey outcomes were described and analyzed on intergroup differences at both time points and intragroup differences over time.

The secondary outcome was FEV_1_ device usage over the 4-month study period. All statistical analyses were performed using Statistical Package for the Social Sciences (SPSS version 25; IBM).

## Results

A total of 39 participants (26 patients and 13 HCPs) were included in this study. Patient characteristics at baseline are summarized in Table 1. HCPs were employed as pediatric pulmonologists (5/13, 38%), general pediatricians (1/13, 8%), residents (2/13, 15%), specialist nurses (4/13, 31%), or doctor’s assistants (1/13, 8%). The median (IQR) age of HCP was 54 years (38.5-58). Baseline lung function was defined as a measurement recorded within 31 days of FEV_1_ device reception. Two patients did not record any lung function measurements and 1 patient did not record a lung function measurement within the 31-day baseline window. One patient quit the study due to loss of interest. During this study none of the patients experienced a PEx nor were hospitalized.

**Table 1:** Baseline^a^ patient characteristics (N=26).

Characteristics			Value
Age (years), median (IQR)			13.4 (11.4-14.6)
**Age group, n (%)**			
	6-11 years	8 (31)	
	12-18 years	18 (69)	
Male, n (%)			12 (46)
Initial ICS^b^ dose µg/day^c^, mean (SD)			488.5 (353.6)
(C-)ACT^d^ score (n=7), median (IQR)			20 (15-23)
ACT^d^ score (n=17), median (IQR)			22 (18-27)
(C-)ACT <20 points (n=21), mean (SD)			8 (38.1)
**6-11 years (n=6)**			
	z-FEV_1_^e^, mean (SD)	–0.30 (0.80)	
	z-FVC^f^, mean (SD)	0.26 (0.50)	
	Tiff (%), median (IQR)	84.08 (73.19-92.00)	
	z-FEF25-75^g^, mean (SD)	–0.22 (1.49)	
	Color zone not green, n (%)	0 (0)	
**12-18 years (n=17)**			
	z-FEV_1_, mean (SD)	–1.53 (1.47)	
	z-FVC, mean (SD)	–0.65 (2.47)	
	Tiff (%), median (IQR)	81.87 (77.84-89.20)	
	z-FEF25-75, mean (SD)	–1.37 (1.07)	
	Color zone not green, n (%)	6 (35)	

^a^Baseline lung function outcomes were defined as the first measurement within 31 days of receiving the FEV_1_ measurement device. Three of the included participants did not perform a baseline lung function measurement.

^b^ICS: inhaled corticosteroids

^c^Beclomethasone or equivalent dose.

^d^(C-)ACT: (Childhood) Asthma Control Test.

^e^FEV_1_: forced expiratory volume in 1 second.

^f^FVC: forced vital capacity.

^g^FEF: forced expiratory flow.

Survey response rates at start were 96% (25/26) for patients and 100% (13/13) for HCPs, whereas those at the end were 85% (22/26) and 92% (12/13), respectively. Survey results and intragroup comparisons over time are summarized in Tables 2 and 3.

A total of 18 (69%) patients used their FEV_1_ device more than 3 times during the study period. FEV_1_ devices were used with a median frequency of 7.5 (IQR 3.3-25.5) distributed over 5.5 unique days (IQR 2.3-19.0). Two patients did not use their FEV_1_ device at all, because one quit the study and another experienced technical difficulties. Most measurements were performed in the morning (154/421, 36.6%) or evening (156/421, 37.1%). 30.6% (129/421) of measurements were not in the personalized green zones, and 11.6% (49/421) of measurements were in the personalized red zone, leading to closer inspection by HCPs.

Seven patients reported that they did not want to decide themselves how often they measured their lung function (Table 2, Q12). Only 2 of these patients used their device with a frequency within the IQR of all participants. The others used their device as follows: not at all, once or twice, or very often (33 or 41 times).

**Table 2:** Patient survey outcomes.

Statement^a^	Start, median (IQR)	End, median (IQR)	Significance (*P* value)
Q1. I am experienced with using smartphones.	5 (4-5)	N/A^c^	—
Q2. Using innovations in health care is normal.	4 (3-5)	N/A	—
Q3. The FEV_1_^b^ measurement device looks nice.	N/A	4 (3-5)	—
Q4. The FEV_1_ measurement device is easy to use.	N/A	4 (2-4)	—
Q5. The manual of the FEV_1_ measurement device was clear.	N/A	4 (3-4)	—
Q6. Measuring my FEV_1_ at home is new for me (innovative).	4 (4-5)	N/A	—
Q7. I should be able to measure my FEV_1_ for a longer period of time.	4 (4-5)	N/A	—
Q8. Measuring my FEV_1_ at home is a good addition to my daily asthma care.	4 (4-5)	4 (4-5)	.83
Q9. The FEV_1_ measurements will provide me with more insights into my disease.	4 (4-5)	4 (3.75-4)	.20
Q10. Measuring my FEV_1_ at home will cost a lot of time.	2 (2-2.5)	2 (2-3)	.23
Q11. I am glad that I am able to check my FEV_1_ by myself.	4 (4-5)	4 (3-4.25)	.34
Q12. I would only measure my FEV_1_ when I experience symptoms.	2 (2-3)	3 (2-4)	.33
Q13. Measuring my FEV_1_ regularly will help me to better handle my disease.	4 (3-5)	4 (3-4)	.13
Q14. The home measurements of FEV_1_ will make me insecure.	2 (1-2)	1.5 (1-2)	.49
Q15. The home measurements of FEV_1_ will give me stress.	2 (1-2)	2 (1-2.25)	.42
Q16. I only want to know my FEV_1_ when it’s not going well.	2 (1.5-2.5)	2 (1-3)	.27
Q17. I want to decide myself how often I measure my lung function.	3 (3-4)	3 (2.75-4)	.86
Q18. I want to receive feedback on my home measurements.	4 (3-4)	4 (3-4.25)	>.99
Q19. I wouldn’t mind to fill out a short symptom survey if my FEV_1_ is lower than expected.	4 (4-5)	4 (4-4)	>.99
Q20. I would like to receive reminders in the online asthma clinic to measure my FEV_1_.	4 (3.5-4.5)	4 (4-5)	.37
Q21. The graphical presentation of my FEV_1_ measurements is useful.	N/A	4 (3-4)	—
Q22. If I don’t succeed to measure my FEV_1_ at home, I know whom to contact.	N/A	4 (4-5)	—
Q23. I don’t worry as long as I feel good, even if my FEV_1_ is lower than expected.	N/A	3 (3-4)	—
Q24. I wouldn’t mind if my health care professionals can see my home measurements.	4 (4-5)	4 (4-5)	.48
Q25. I would feel controlled by my health care professionals if they can see my home measurements.	2 (1-3)	2 (1-2.25)	.21

^a^Responses were collected on a 5-point Likert scale, where 1=strongly disagree; 2=disagree; 3=neutral; 4=agree; and 5=strongly agree.

^b^FEV_1_: forced expiratory volume in 1 second.

^c^N/A: not applicable.

**Table 3:** Health care professional survey outcomes.

Statement^a^	Start, median (IQR)	End, median (IQR)	Significance (*P* value)
Q1. I am experienced with using smartphones.	4 (3.5-4.5)	4 (4-4.75)	>.99
Q2. Using innovations in health care is normal.	4 (4-5)	4 (4-5)	>.99
Q3. The FEV_1_^b^ measurement device looks nice.	N/A^c^	4 (4-4.75)	—
Q4. The FEV_1_ measurement device is easy to use.	N/A	4 (3-4)	—
Q5. The manual of the FEV_1_ measurement device was clear.	N/A	4 (4-4)	—
Q6. Letting patients measure their FEV_1_ at home is innovative.	4 (4-5)	N/A	—
Q7. Patients should be able to measure their FEV_1_ at home for a long period of time.	4 (4-5)	N/A	—
Q8. Home monitoring of FEV_1_ is a good addition to patients’ daily asthma care.	4 (4-5)	4 (4-5)	.77
Q9. The graphical presentation of the FEV_1_ measurements is useful.	N/A	4 (4-5)	—
Q10. The FEV_1_ measurements will provide me with more insights into my patients’ disease.	4 (4-4.5)	4 (4-4)	.48
Q11. I only want patients to measure their FEV_1_ when they experience symptoms.	2 (2-3)	2 (2-2.75)	>.99
Q12. Possible deteriorations will be detected earlier thanks to the home measurements.	4 (4-4.5)	4 (4-4.75)	.74
Q13. If technical problems arise with the measurements, I know whom to contact.	4 (3.5-4)	4 (3.25-4.75)	.48
Q14. The FEV_1_ measurements will pose an additional time burden for me.	3 (3-4)	3 (2-3)	.02^d^
Q15. I only want to know the FEV_1_ measurements when they are lower than expected.	4 (2.5-4)	4 (2.25-4)	.41
Q16. Patients themselves should be responsible about how often they measure their FEV_1_.	3 (2-3.5)	3.5 (3-4)	.84
Q17. If FEV_1_ measurements are lower than expected, a short symptom survey will provide sufficient information for me.	3 (3-4)	4 (3.25-4)	.10
Q18. Patients should receive reminders in the online asthma clinic to measure their FEV_1_.	4 (3-4)	4 (2.25-4)	.10
Q19. I know which patients are eligible for home monitoring of FEV_1_.	4 (4-4)	4 (3.25-4.75)	.32

^a^Responses were collected on a 5-point Likert scale, where 1=strongly disagree; 2=disagree; 3=neutral; 4=agree; 5=strongly agree.

^b^FEV_1_: forced expiratory volume in 1 second.

^c^N/A: not applicable.

^d^Statistically significant change between survey outcomes at end and start.

Patients were interested in knowing all of their measured FEV_1_ values. By contrast, HCPs at both start and study end were more interested in only knowing their patients’ FEV_1_ outcomes when they were lower than expected (*P*=.006 and .001, respectively). At the start, HCPs were significantly more likely to expect an increased time burden compared with patients (*P*<.001). However their expectations were not matched by their experiences because at study end HCPs had significantly improved opinions on time burden compared with study start (*P*=.021; Table 3, Q14). Reported benefits, disadvantages, and suggestions by HCPs and patients are summarized in Textboxes 1 and 2.

**Patients reported benefits, disadvantages, and suggestions at the end of the study. FEV_1_: forced expiratory volume in 1 second.**

**Benefits**

- More insights into my/my child’s asthma
- Improved asthma control
- Less frequent hospital visits
- Always being able to know how I am/my child is doing
- It helps me with my/my child’s disease perception
- More insights into my/my child’s lung function
- Facilitates easy and quick adjustment of treatment
- Signaling of low lung function

**Disadvantages**

- Wrong values when the measurement is performed incorrectly
- The device does not always work
- It is easy to lose the device
- It is easy to forget to perform measurements
- The FEV_1_ device is less reliable than the device in the hospital
- Performing the FEV_1_ measurements is time consuming
- The mouthpiece is too large for small children

**Suggestions**

- Reminders should be sent to perform an FEV_1_ measurement
- The FEV_1_ device should be easier to use
- Instructions on how to clean the device
- Smaller mouthpiece for smaller children

**Health care professionals’ reported benefits, disadvantages, and suggestions at the end of the study. FEV_1_: forced expiratory volume in 1 second.**

**Benefits**

- Provision of objective measures of asthma control
- Aids patients with disease perception
- Continuity of monitoring
- Earlier recognition of pulmonary exacerbations
- Taking a simple and quick lung function test is possible when needed
- Facilitates easy and quick adjustment of treatment

**Disadvantages**

- More stress for both patients and caregivers
- More emphasis on patients’ asthma
- FEV_1_ measurements might be performed incorrectly which can falsely comfort or alarm patients
- Compulsive FEV_1_ testing and less attention for perceived symptoms
- Decreased motivation when FEV_1_ remains low while symptoms are barely present
- Time consuming for both patients and health care professionals
- Technological difficulties of FEV_1_ devices

**Suggestions**

- Good instructions at baseline
- FEV_1_ devices should be calibrated at every outpatient visit
- FEV_1_ home measurements should be used as a means to aid patients in achieving their individual goals. FEV_1_ home measurements should not be a goal on their own
- Better instructions for patients on what to do when their FEV_1_ is lower than expected
- Less notifications for health care professionals during pulmonary exacerbations
- A notification when patients are back in their green zone
- Protective case for a fragile FEV_1_ device

## Discussion

Our findings show that both patients/their parents and HCPs are receptive toward online FEV_1_ telemonitoring in pediatric asthma care. The participants in this study agreed that FEV_1_ telemonitoring in pediatric asthma care is innovative and improves asthma care, self-management, and disease perception. Most of the expectations patients and HCPs had about FEV_1_ telemonitoring matched their experiences. Initially, HCPs had concerns regarding the additional time burden and increased stress and insecurities of patients. However, HCPs’ experienced time burden was eventually lower than expected and increased stress and insecurities of patients were not reflected in the patients’ own experiences.

In our self-paced monitoring protocol, 69% (18/26) of patients used their FEV_1_ device regularly, which affirms survey-reported receptiveness of patients. However, the large interindividual range in the frequency of use between patients emphasizes the different approaches of individuals. Only 11.6% (49/421) of measurements were in the “red zone.” The vast majority of measurements required no intervention or could be resolved with automatic feedback prompts using patients’ personalized online asthma action plans.

To our knowledge, this is the first study on patients’ and HCPs’ receptiveness of FEV_1_ home monitoring combined with an online eHealth platform in pediatric asthma care. The existing body of literature often reports acceptance of FEV_1_ home monitoring, but it is rarely quantified [12-15]. Because of the COVID-19 pandemic, there is an increased call for research into user satisfaction and receptiveness of eHealth interventions [8,9]. Before the COVID-19 pandemic, Simpson et al [23] surveyed 187 patients with asthma and 63 HCPs on eHealth interventions and found that both groups supported the use of eHealth and preferred eHealth over paper diaries and paper asthma action plans. They also found that in both groups lung function measurements were expected to be the most contributive additional measurement for asthma control in an eHealth program [23]. This highlights the increasing demand for home monitoring of lung function among patients. In our small population, expectations of both patients and HCPs were matched by their experiences, underlining that patients and their parents can assess for themselves whether FEV_1_ telemonitoring will aid them. Therefore, we argue that patients interested in the ability of online FEV_1_ monitoring should receive this opportunity to assess personal benefits.

How patients achieve personal benefits should be a result of shared decision making between patients and their HCPs. We now know that standalone FEV_1_ monitoring with strict monitoring regimes in most patients does not work out [12-15]. By contrast, we believe that in the future FEV_1_ devices should be used to reach personalized goals, such as reassurance, as an aid to improve symptom perception or to quantify lung function during episodes with increased symptoms. When integrated into an eHealth platform, these devices can be used at the patient’s convenience and provide immediate feedback. It is our opinion that these set ups can facilitate these goals more easily and quickly than standalone FEV_1_ monitoring. The results of our study support this opinion. In this study most patients wanted to keep their own responsibility on how often they measured their lung function. Subsequently, we found a large variation in frequency of use between participants, but there was a consensus nonetheless on the perceived benefits of asthma control and disease perception by both patients and HCPs. Some patients did not want to keep the responsibility on how often they measured their lung function and as a result most of these patients either rarely used the devices or overused them. This also shows that for some patients a structured monitoring regime could be indicated. Self-management depends on several factors such as individual health skills, disease perception, beliefs, and the interaction between patients and their HCP, and thus one size does not fit all. To ensure longevity and efficiency of FEV_1_ telemonitoring, a personalized approach should be used: patients who want to keep the responsibility to themselves should be offered this chance, whereas those who do not want to should be offered a schedule. To better understand this personalized approach, it is important that both the clinical and perceived benefits of the different approaches are studied experimentally in more detail. It is important to identify which patients benefit most from which intervention and how we define personal benefits.

Recurring concerns of FEV_1_ telemonitoring are toward the reliability of the measurements. These were also present in our population and are generally shared among the scientific community [1,4,24,25]. Although perfect correlation of FEV_1_ home measurements with hospital measurements is desirable, one can argue that FEV_1_ monitoring will primarily be used to ascertain an FEV_1_ above a certain threshold. In that case a somewhat lower FEV_1_ may not be of any clinical relevance. By contrast, invalidly low FEV_1_ values at home may lead to excessive health care consumption, medication use, and disease-related stress. Online FEV_1_ home monitoring should function as an aid for symptom perception and a quick objective measure during symptoms. In our opinion these aims do not require an FEV_1_ outcome as sensitive as that measured by a spirometer operated by a specialist nurse. Nevertheless, reliability should be studied in more detail to rule out counterproductive measurements.

This study was limited by its small study population and short study duration, which were chosen due to the nature of this viewpoint study. Only including patients and HCPs from specialized asthma clinics who also had experience using our eHealth platform would have introduced selection bias. In our opinion this does not invalidate our findings; by selecting patients and HCPs who already have experience with the eHealth platform we avoid confounding of our qualitative outcomes by the introduction of the eHealth platform. We also still observed a large interindividual variation in FEV_1_ device usage. Finally we believe that home monitoring strategies like this will be reliant on some form of selection bias as they should primarily be applied in patients that are receptive. This might imply that our results are not extrapolatable to settings without previous eHealth experience, general practitioner services, or to low-resource settings. Furthermore, we performed no post measurement quality control of the FEV_1_ measurements, which may have led to technically incorrect measurements being recorded. However, the software of the FEV_1_ device performs a quality check based on the American Thoracic Society (ATS)/European Respiratory Society (ERS) consensus statement [26,27].

### Viewpoint

Our findings show that patients and HCPs are receptive toward online FEV_1_ home monitoring combined with an online eHealth platform. Frequency of measurements varies largely among individuals, yet perceived benefits remain similar. This emphasizes that online FEV_1_ home monitoring strategies should be used as a means to reach individual goals, rather than being a goal on their own. During the COVID-19 pandemic we have seen eHealth being integrated more into daily medical practice and this will undoubtedly increase even more in the near future. With this increasing use of eHealth, we will see an equal surge of different ways to monitor our patients. In this data tangle it is easy to lose sight of what really matters: patient quality of life and needs. If we want to continuously monitor our patients, we should not only study the clinical relevance, but also the experienced benefits, invasiveness, and perceptions of our patients, while keeping in mind that one size does not fit all. Even after implementation we should repeatedly monitor patient satisfaction. eHealth is a dynamic entity and strongly bound to technologic advances. Patients should have a say in how these advances will be utilized and in which way they add value to their lives. HCPs have to remember that the goal should not be to gather outcomes, but to use these outcomes to reach personal goals in a personalized way.

## Abbreviations

ACT: Asthma Control Test
ATS: American Thoracic Society
(C-)ACT: (Childhood) Asthma Control Test
ERS: European Respiratory Society
FEF: forced expiratory flow
FEV_1_: forced expiratory volume in 1 second
FVC: forced vital capacity
GINA: Global Initiative for Asthma
HCP: health care professional
PEx: pulmonary exacerbation
